# Is the disparity in perinatal mental health services dependent on race? A narrative review. “A race to access”

**DOI:** 10.1192/j.eurpsy.2022.2212

**Published:** 2022-09-01

**Authors:** T. Awolesi

**Affiliations:** University of Bristol medical school, Faculty Of Health Sciences, Bristol, United Kingdom

**Keywords:** perinatal mental health, Ethnicities, disparity, maternal health

## Abstract

**Introduction:**

Today the maternal death of black women is four times than the maternal death of white women. A lot has been written about the physical health of black women during pregnancy and childbirth however the perinatal mental health of this group of women is less well researched. I wanted to investigate if black and ethnic minority women in the UK had the same access to perinatal mental health services.

**Objectives:**

To explore how the access to perinatal mental health services vary between white British and non-white British women.

**Methods:**

A literature review was conducted. Papers were selected based on their focus on perinatal mental health service access and differences in access based on ethnicities. Most research focused on the perinatal mental health service access of white British and non-white British groups of women.

**Results:**

The literature review revealed that black African, Asian and minority white women had significantly lower access to community perinatal mental health services when compared to white British women. It was also found that that black African, Asian and minority White women had a higher percentage of involuntary admissions to psychiatric hospitals when compared to white British women.

**Conclusions:**

The literature would suggest that there is less access to perinatal mental health for non-white British women. This suggested that the disparities that exist within perinatal physical health extend into perinatal maternal health.

**Disclosure:**

No significant relationships.

